# Impacts of human recreation on carnivores in protected areas

**DOI:** 10.1371/journal.pone.0195436

**Published:** 2018-04-05

**Authors:** Angela Darnell Baker, Paul L. Leberg

**Affiliations:** Department of Biology, University of Louisiana at Lafayette, Lafayette, Louisiana, United States of America; Michigan State University, UNITED STATES

## Abstract

Mammalian carnivores can be particularly sensitive to human disturbance, even within protected areas (PAs). Our objective was to understand how human disturbance affects carnivore communities in southern Arizona, USA by studying habitat occupancy based on data collected using non-invasive methods in three PAs with different levels of human disturbance. Carnivore occupancy varied based on human disturbance variables (i.e., roads, trails, etc.). Common carnivore species (coyotes, gray foxes, and bobcats) had high occupancy probability in highly disturbed sites, while all other carnivore species had a higher probability of occupancy in low disturbance protected areas. Additionally, overall carnivore diversity was higher in PAs with low human disturbance. Edges of PAs appeared to negatively impact occupancy of nearly all carnivore species. We also found the presence of roads and trails, and not necessarily how much they are used, had a significant negative impact on the occupancy of most carnivore species. Furthermore, the overall level of disturbance within a PA influenced how sensitive carnivores were to human disturbance variables. Carnivores were more sensitive in PAs with higher levels of disturbance and were relatively unaffected by disturbance variables in a PA with low base levels of disturbance. Increased visitation to PAs, expected with the region’s high level of population growth, is likely to cause shifts in the carnivore communities favoring species that are less sensitive to disturbance.

## Introduction

Mammalian carnivores are often a vital component of ecosystems, influencing community structure, stability, and diversity [1–4]. They can also be important surrogate species for conservation priorities, acting as indicators of biodiversity, umbrella species, or flagship species in their respective ecosystems [5–7]. Human disturbance has been shown to affect the diversity, composition, and structure of many communities [8–10], and carnivores are particularly sensitive to human disturbance due to their relatively large body sizes, large home ranges, low fecundity, long generation times, and low populations densities [2, 11, 12]. Adding to their vulnerability is the fact that they often come into conflict with humans [13]. Cardillo, Purvis [14] found that intrinsic biological factors, such as those mentioned above, interact with human population density to account for a large portion of the extinction risk of carnivores. Thus, in many parts of the world, protected areas (PAs) are the last available refuge for carnivore species and are essential to their persistence [11, 14, 15].

Margules and Pressey [16] contend that one of the primary roles of a PA is to separate the diversity of organisms within it from the activities that threaten their existence. We considered a PA as an area dedicated to and managed for the “…conservation of nature with associated ecosystem services and cultural values” [17]. Examples of such areas in the United States would include state and national parks and forests, conservation easements, and wildlife refuges. Although carnivores in these PAs may not face hunting mortality, they are not immune to the impacts of human disturbance [18, 19]. Most PAs allow access for hiking, camping, and other recreational activities, all of which can have significant impacts on wildlife [20–22]. Reed and Merenlender [23] found that quiet, non-consumptive recreational activities (e.g., hiking) resulted in a significant decrease in density (5x fewer) of native carnivores compared to PAs that were not open to the public. A recent review found that 59% of studies documented negative effects of recreation in protected areas on wildlife [24]. As the impacts on carnivore communities may have far reaching impacts on ecosystems, determining the effects of human disturbance in PAs is therefore essential for improving science-based conservation policies [15, 25, 26].

The carnivore community of the southwestern United States has been relatively understudied. Although there are published studies that focus on individual or small subsets of species (e.g., [27],[28]), examinations of the broader community are lacking [29]. To date, there are no studies from this region that examine the impacts of human disturbance on whole carnivore communities within PAs, despite such information being necessary to effectively protect both carnivores and the ecosystems in which they function [25]. Carroll, Noss [30] suggest that a sufficient conservation plan for carnivores must account for the requirements of multiple species which might have conflicting habitat requirements, which highlights the need for studies of entire carnivore communities to adequately aid conservation efforts. Additionally, protected areas along the U.S.-Mexico border face a number of unique challenges associated with migrants and drug smugglers that could affect carnivore management. For example, movements of these individuals as well as associated enforcement activities might create more nocturnal disturbances than would occur in other PAs. Also, activities from such individuals often occur in remote areas not often visited by recreationists (P. Holm, NPS, personal communication).

In recent years, annual visitation rates to national parks and other PAs have increased significantly [31, 32]. For example, visitation to Saguaro National Park has increased 103% in the last 5 years [32]. Increased human disturbance due to traffic and the addition of new infrastructure generally accompanies increases in visitation. Roads and other infrastructure can fragment habitat. Nee and May [33] suggest that habitat loss can modify the composition of communities in remaining habitat, even when the remaining habitat does not experience any fundamental changes. To adequately conserve carnivore species within PAs, managers need information on how each species within the community responds to different types and intensities of anthropogenic disturbance.

Our objective was to examine carnivore occupancy with respect to human disturbance variables within PAs that receive varying levels of disturbance. We hypothesized that the level of human disturbance within a PA would impact carnivore habitat occupancies, and that there would be species-specific responses to disturbance variables. We predicted that higher levels of disturbance within a PA would negatively impact occupancies of carnivore species and that species which are most sensitive to human activity will have lower occupancy in areas of high human disturbance.

## Material and methods

All appropriate ethics and other approvals were obtained for the research. The research protocols were approved by an authorized animal care and use committee of the University of Louisiana at Lafayette (IACUC nos. 2014-8717-025 and 2015-8717-018). Permits for field work done in Saguaro National Park and Organ Pipe Cactus National Monument were obtained from the National Park Service.

### Study area

**Fig 1:**
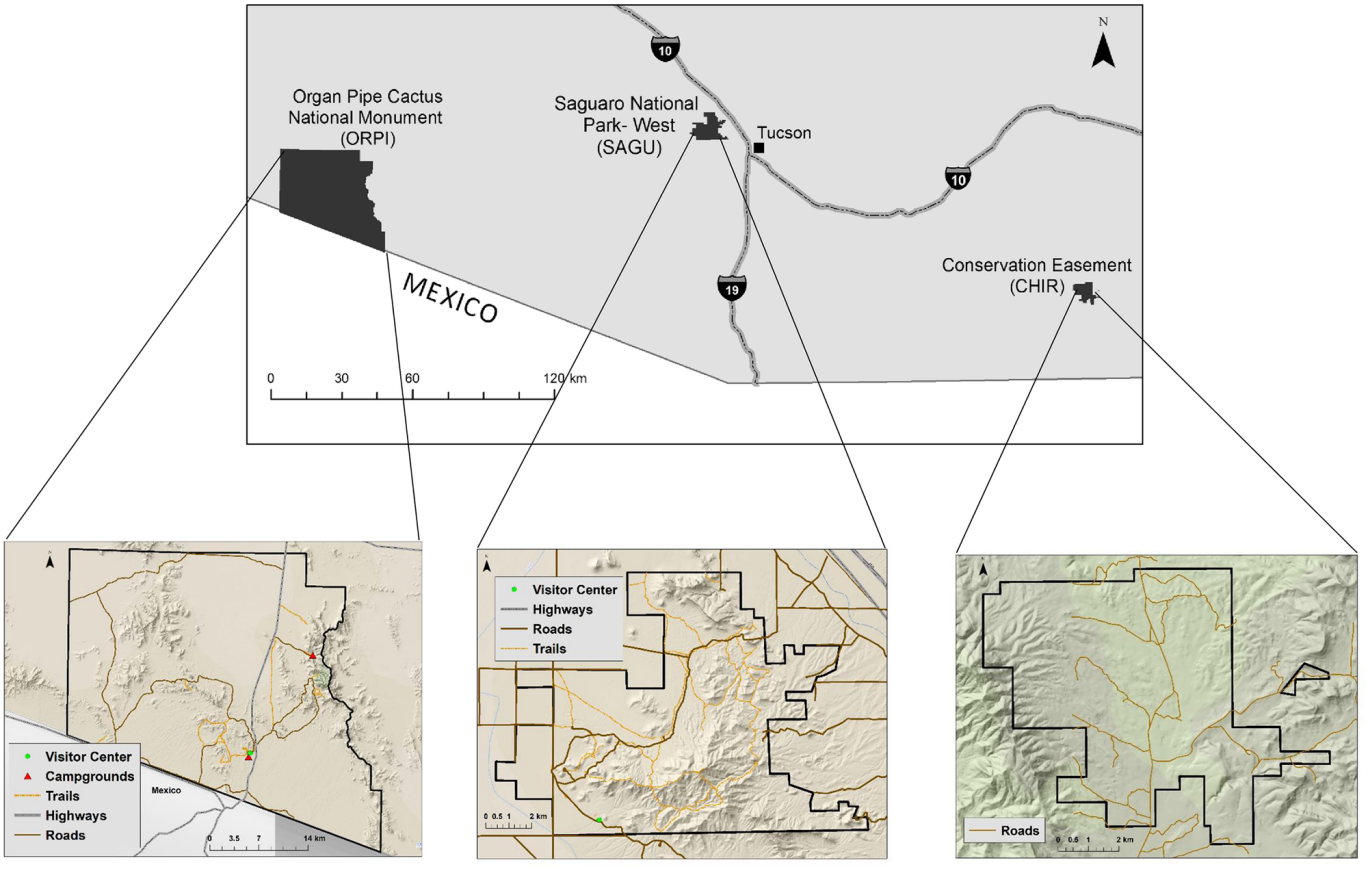
Map of study area in southern Arizona showing the locations of the 3 protected areas.

We conducted our study in three PAs: private property in the foothills of the Chiricahua Mountains (CHIR), Organ Pipe Cactus National Monument (ORPI), and Saguaro National Park (SAGU), all located in southern Arizona (Fig 1). None of these areas allow hunting or other extractive use and all contained a similar complement of carnivores (up to 15 species; [34, 35]). There were no significant differences in management activities between the three sites as they are all managed under federal guidelines (National Park Service and U.S. Fish and Wildlife Service).

Our low disturbance site, CHIR, is a 58.27 km^2^ private ranch property (31.61° N, -109.48° W) that has no public visitors or hiking trails, and all of the roads on the property (0.15 km/km^2^) are unpaved. The ranch is managed as a Conservation Easement with the U.S. Fish and Wildlife Service, north of the Leslie Canyon National Wildlife Refuge and adjacent to the Coronado National Forest, which lies to the east. The other surrounding areas are used for private livestock ranching. Elevations range from 1400 to 2400 m. The property supports a small number of cattle (100–150) which are periodically rotated throughout the property. Carnivore home ranges likely overlapped with livestock on the property, and this was accounted for in our human disturbance index (see methods below). Due to its location near the border of Mexico as well as its relative isolation from human development, it was occasionally visited by trans-border migrants and drug smugglers (personal observation).

We considered ORPI (31.95°N, 112.80°W) an area with a moderate level of human disturbance (1.18 million annual visitors; [32]). The National Park Service (NPS) administers the 1,332 km^2^ monument which, while containing a very low density of roads (0.03 km/km^2^ of paved roads and 0.08 km/km^2^ of unpaved roads), does contain a state highway that runs through the park. ORPI also has a low density of 0.04 km/km^2^ of hiking trails. The monument is within the Sonoran Desert and consists of extensive alluvial basins around 300 m separated by steep mountains up to 1460 m [34]. As ORPI is adjacent to the Mexican border on the south, this area is often heavily used by trans-border migrants and drug smugglers (personal observation; P. Holm, NPS, personal communication). The west and part of the north sides of the park are bordered by the Cabeza Prieta National Wildlife Refuge; the rest of the north is adjacent Bureau of Land Management land. The east is bordered by the Tohono O’odham National Reservation.

Saguaro National Park (32.25° N, 111.16° W), also administered by the NPS, was considered a site with a high level of human disturbance (3.74 million annual visitors; [32]). The study sites were exclusively in the Tucson Mountain District (TMD), which is just west of the city of Tucson and surrounded by development. The TMD is 99.15 km^2^ and part of the Sonoran Desert ecosystem. The park contains a relatively high density of roads (0.27 km/km^2^ paved and 0.16 km/km^2^ unpaved) as well as 0.86 km/km^2^ of hiking trails. The elevation of TMD ranges from 664 to 1429 m [35].

Vegetation within the PAs varies by elevation. The desert floors are dominated by cacti (25 different species) including saguaro (*Carnegiea gigantean*) and several species of *Optunia* spp. (prickly pear and chollas; [34, 35]). Small trees and shrubs are also common including: creosote (*Larrea tridentata*), palo verde (*Parkinsonia* spp.), mesquite (*Prosopis* spp.), acacia (*Acacia* spp.), and ironwood (*Olneya tesota*; [36, 37]). The Lower Sonoran zone consists of arid grasslands in the lower-mid elevation areas (up to 1500 m), where mesquite, acacia, agave (*Agave* spp.), and yucca (*Yucca* spp.) are common [37]. The higher elevations of the Upper Sonoran zone (1500–2500 m) contain abundant oaks (*Quercus* spp.) and junipers (*Juniperus* spp.; [37]). In the desert valleys, summer temperatures range from 18–40°C, while winters are between 4–22°C [38]. In the higher elevations, summers temperatures are lower, 13–33°C, while winters are considerably cooler, -4-13°C [38]. Precipitation peaks during summer monsoon rains, ranging from 23–27 cm in the desert basins and 45–79 cm at higher elevations [38]. The species and genera of potential prey were similar between sites (Baker, unpublished data). With the exception of the border fence adjacent to the south of ORPI, fences in both NPS sites and in CHIR were simple consisting of 1.2–1.5 m high metal or wooden posts with wire strung widely (30–45 cm) between them; these types of fences are unlikely to significantly hinder wildlife movement (personal observation).

### Data collection

As carnivores are elusive and the probability of detecting different species can vary based on method [29, 39], we used several non-invasive techniques including: remote cameras, track plates (covered and uncovered), and natural sign surveys. We conducted surveys between Aug 2014 and July 2015 at 40 sites at least 1 km apart in random locations (selected using a random point generator in ArcGIS v10.2) in each PA. One remotely triggered passive infrared trail camera (Moultrie M-880i) and four track plates (2 covered and 2 uncovered) were placed with bait (a spoonful of canned cat food) in each survey site for 8 days, Based on a pilot study of different baits, cat food was an effective bait for desert carnivores as it attracts a variety of species, and its scent remains despite the arid environment (Baker, unpublished data). Track plates and cameras were re-baited every 2 days. Cameras were placed 0.25–0.5 m from the ground [40] and operated 24 hours a day with a 30 second delay. We conducted natural sign surveys every other day, concurrent with the deployment of the cameras and track-plates. These surveys consisted of systematically walking around the survey location within a 50 m radius, attempting to cover the entire 785 m^2^ area, looking for scat, tracks, burrows or other signs of carnivores. All signs were either collected (i.e., scat) or otherwise removed (i.e., tracks) except for burrows and dens (which were only counted if they were active). Because burrows could not be removed, they were only counted on the first survey at each site, unless it was obvious that one had been freshly dug (confirmed by author, ADB). To reduce observer bias, field technicians were rotated between sites each day. All observers took pictures of each sign, and ADB verified all identifications. If a positive ID could not be made, we discarded the entry (< 5% of signs). Although PAs were different in size, limiting our sampling to 40–785 m^2^ sites meant that each PA had 0.03 km^2^ of area surveyed.

Track-plates were constructed according to Zielinski and Kucera [41] and Long, Mackay [40]. Plates were made of sheets of aluminum, 20 cm x 61 cm x 0.12 cm for closed plates and 61 cm square for open plates. Closed plates were surrounded by white, corrugated plastic boxes and backed by pieces of tarp. We coated the plates with powdered carbon and placed bait in the back (closed) or middle (open) of the plate. We identified tracks, re-baited, and re-coated the carbon every 2 days.

To account for seasonal variation in occupancy and detection rates, we resurveyed locations each season (summer, fall, winter, spring; [42]); thus, each site was surveyed for 8 days, once per season. Possible confounding variables between sites were recorded including: temperature, precipitation, elevation, and major vegetation type. Additionally, we determined the distance to nearest human infrastructure, road, trail, and edge of PA using ArcGIS. Distance from infrastructure ranged from 120–32,802 m ($\bar{x}=5176.4$ SD 7178.9), from roads ranged from 3.5–3777 m ($\bar{x}=575.4$ SD 549.7), from trails ranged from 8–25,172 m ($\bar{x}=5746.1$ SD 3418.9), and from edge of PA ranged from 50–15,852 m ($\bar{x}=3628.1$ SD 4082.9). We obtained information on the number of visitors in each park as well as road and trail use using the NPS Visitor Use Statistics and county data. All trails were used only by hikers and horse-back riders; wheeled vehicles were prohibited (including mountain bikes, motorbikes, and ATVs). We also obtained information on Border Patrol (BP) activity at each of our sites in ORPI, where BP presence is heavy (P. Holm, NPS). Detailed descriptions for each variable including the calculation of human disturbance index can be found in Supplemental Materials.

### Data analysis

We used occupancy modeling to estimate the occupancy and detection probabilities of each carnivore species. When detection probabilities are less than one, the proportion of sites in which a species is detected will always underestimate the actual level of occupancy [43]. Occupancy modeling utilizes detection histories from repeated observations to estimate true occupancy levels. The models allow occupancy to vary according to site characteristics (e.g., elevation, human disturbance variables), and detection probabilities to vary with survey characteristics (e.g., precipitation). We counted a species as present for each survey if it was detected during a sign survey, on a track plate, or was observed on a camera in the first 24 hours following the time it was baited/re-baited.

Detection histories of each carnivore along with site and survey covariates were input into the program PRESENCE v 10.7 [44]. Carnivores needed to be detected in at least 10% of the sample sites in a single PA to be included in the models [29]. Using this program, we estimated occupancy (psi) and detection probability (p) of each carnivore species. All occupancies reported refer to the probability that a given site is occupied (psi). Occupancy and detection probabilities were determined using maximum likelihood estimation methods [44]. Models included a base model where detection probability and site occupancy are considered constant, and several models in which detection and occupancy varied with covariates. Models were ranked according to Akaike’s Information Criterion adjusted for small sample sizes (AIC_c_) and Akaike weights (*w*_*i*_; [45]). We also estimated the over-dispersion parameter ĉ of the global model for each species, using the parametric bootstrap method in PRESENCE (1000 simulations). If the model was over-dispersed (ĉ was >1), we inflated the standard errors by a factor of √ĉ and used the quasi-corrected AIC_c_ (QAIC_c_) to determine model selection [45, 46].

Occupancy models assume that survey sites are independent, however some of the larger carnivores in our study may have travelled further than 1 km in a night. Thus, we first compared the results of the model within PRESENCE that incorporates spatial-autocorrelation of sample sites (using a first-order Markov process) to the standard model using both the null and global models of each species [47]. In all cases, the basic model performed better (lower AIC) and was used for all further analyses.

Covariates related to detection probability included: precipitation, season, and a survey-specific human disturbance index (HD; see Supplemental Information for details). The “across PA” covariates potentially influencing occupancy included: human disturbance index; road and trail use; distance to nearest human infrastructure, edge of PA, road, and trail; season; and park (a dummy variable that differentiates between the three PAs). We could not include area of PA as a covariate, as it was confounded with “park.” We also included PA specific covariates: number of visitors, elevation, vegetation type, and in models for ORPI, an index of Border Patrol activity (low, medium, high). All continuous covariates were standardized using z-scores. For categorical covariates, if there was an inherent order (e.g., road use), we created an ordinal categorical covariate, for example 1 = low, 2 = medium, and 3 = high [48]. For covariates without a natural order (e.g., season), we transformed the variable into a series of binary indicator variables. Correlations between covariates are presented in supplementary materials.

We first used the AIC_c_ of the models to determine which covariates influenced detection probability (*p*) of each species, while holding occupancy constant. We then kept detection probability covariates constant in subsequent models for occupancy [49, 50]. The first model for each species was a global model that included all non-correlated potential covariates and interactions. We then developed a set of candidate models using all combinations of covariates that had a p-value < 0.1 in the global model [51]. We used model-averaged estimates of all parameters [45].

In addition to single species analyses, we also combined detection histories of species considered either common (seen in at least one sample site every season in all PAs) or rare (all other cases) to look at broader patterns of human disturbance effects on carnivores. We determined the diversity of carnivores in each PA using the total number of carnivore species detected within the area each season. Differences among PAs were assessed using ANOVA, blocked by season, and Tukey post-hoc test. All of the above statistical analyses were conducted in R. Statistical tests were considered significant at alpha of 0.05.

## Results

We conducted a total of 1,896 surveys in 120 sample sites (40 per PA). Due to logistic constraints, the SAGU summer surveys only included 34 sites. Thirteen of the potential 15 resident carnivore species were detected using at least one of the survey methods; we had no detections of raccoons or long-tailed weasels. Although hog-nosed skunks (*Conepatus leuconotus*) and white-nosed coatis (*Nasua narica*) were both detected in CHIR, we did not have enough detections to include them in analyses. Gray foxes (*Urocyon cinereoargenteus*) had the highest overall occupancy (psi) within the study area (0.735 ± 0.209), followed by coyotes (0.721 ± .210), and bobcats (*Lynx rufus*; 0.532 ± 0.142). Hooded skunks (*Mephitis macroura*; 0.106 ± 0.12), spotted skunks (*Spilogale gracilis*; 0.113 ± 0.029), and ringtails (*Bassariscus astutus*; 0.133 ± 0.085) had the lowest occupancies of any of the carnivores.

### Carnivore occupancy between PAs

**Fig 2:**
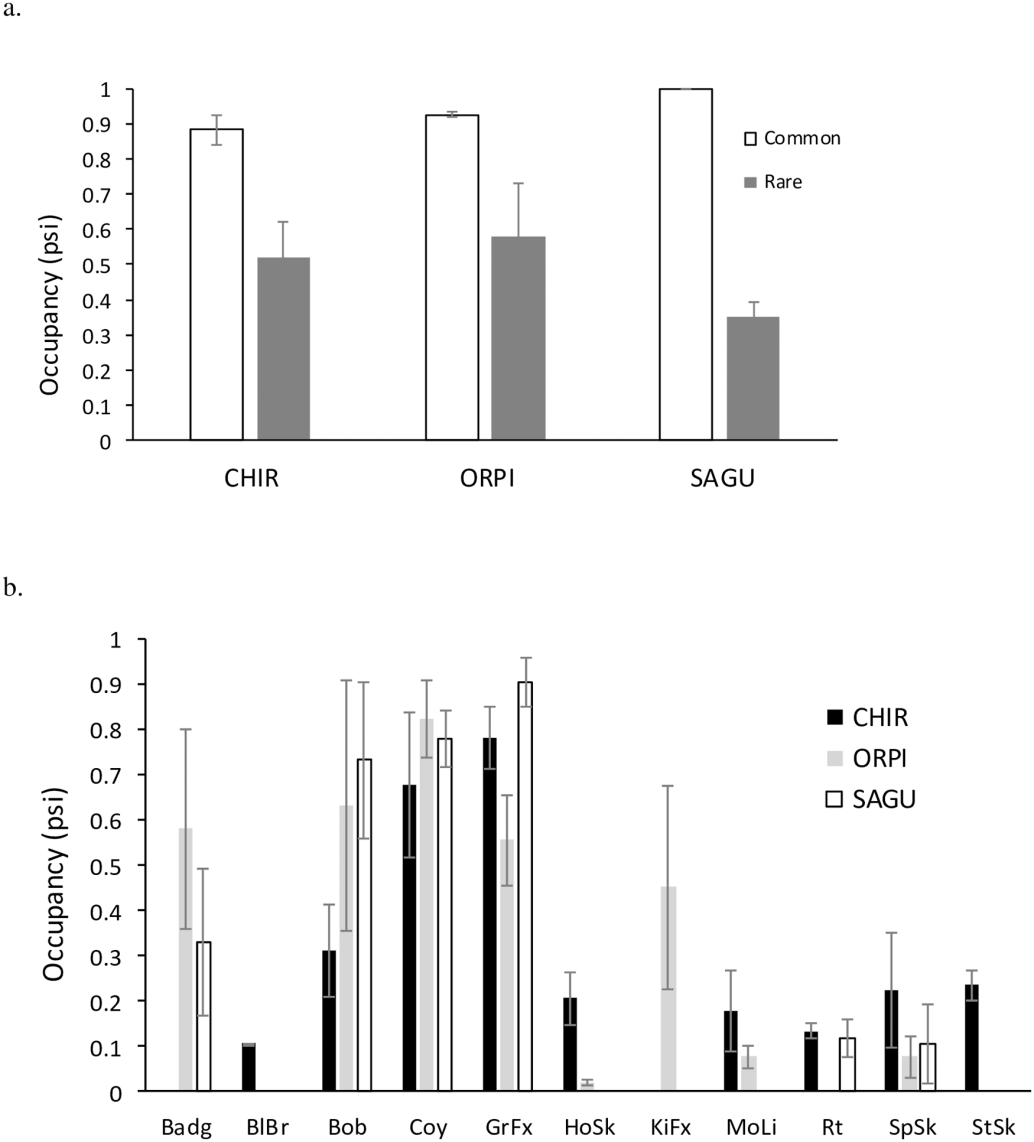
Occupancy (±SE) of a) common and rare carnivores and b) 11 individual carnivore species in 3 protected areas of southern Arizona, 2014–2015. Species abbreviations: Badg = badger, BlBr = black bear, Bob = bobcat, Coy = coyote, GrFx = gray fox, HoSk = hooded skunk, KiFx = kit fox, MoLi = mountain lion, Rt = ringtail, SpSk = spotted skunk, StSk = striped skunk.

Total number of carnivore species detected (species diversity) was significantly lower in SAGU compared to ORPI and CHIR (F_11_ = 10.32, p = 0.005). Occupancy of common carnivore species (coyote, gray fox, and bobcat) was highest in SAGU (1.000 [95% CI = 1.000, 1.000]), followed by ORPI (0.928 [0.912, 0.944]), and lowest in CHIR (0.885 [0.877, 0.893]; Fig 2a). Conversely, occupancy of rare carnivore species was significantly higher in CHIR (0.518 [0.494, 0.542]) and ORPI (0.579 [0.474, 0.684]) compared to SAGU (0.348 [0.261, 0.435]; Fig 2a).

Carnivore species occupancy varied significantly between PAs (Fig 2b). Badger (*Taxidea taxus*) occupancy was significantly higher in ORPI (0.580 [0.534, 0.623]) than SAGU (0.329 [0.209, 0.449]). Occupancy of bobcats was highest in SAGU (0.734 [0.670, 0.798]) and lowest in CHIR (0.331 [0.295, 0.327]). Coyote occupancy was lower in CHIR (0.679 [0.669, 0.689]) than ORPI (0.824 [0.712, 0.936]) or SAGU (0.779 [0.698, 0.860]). Gray fox occupancy was highest in SAGU (0.906 [0.862, 0.951]) and lowest in ORPI (0.555 [0.355, 0.755]) and CHIR (0.782 [0.716, 0.854]). Hooded skunk occupancy was higher in CHIR (0.205 [0.089, 0.322]) than ORPI (0.018 [0.006, 0.030]), and the same was true for mountain lions (0.179 [0.161, 0.197] in CHIR and 0.076 [0.055, 0.097] in ORPI). Occupancy of ringtails was not significantly different between PAs. Spotted skunk occupancy was lower in ORPI (0.076 [0.017, 0.169]) and SAGU (0.104 [0.087, 0.121]) compared to CHIR (0.223 [0.170, 0.276]).

### Human disturbance variables

**Fig 3:**
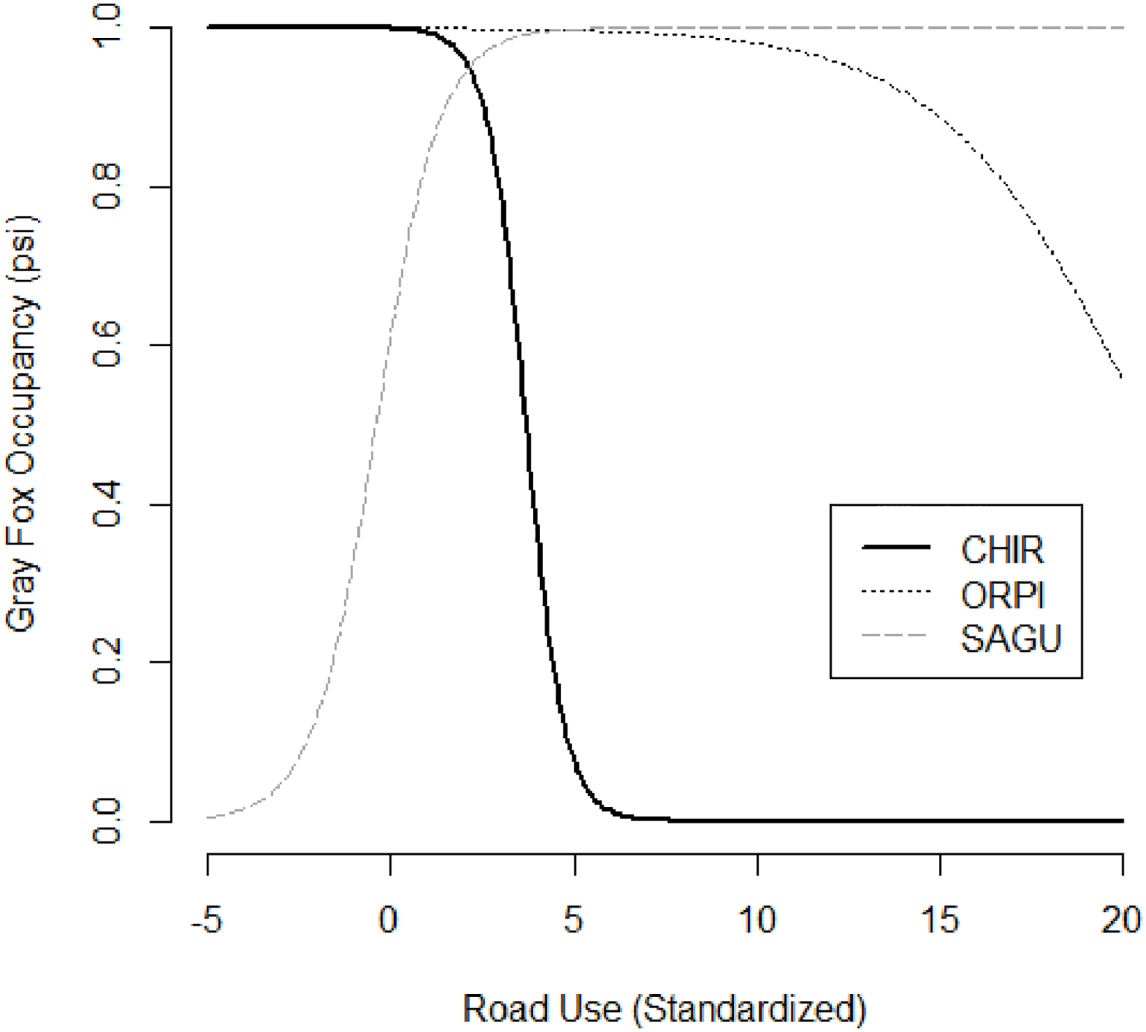
Relationship of gray fox occupancy to road use in 3 protected areas of southern Arizona, 2014–2015.

The top models for each carnivore species had unique combinations of human disturbance and habitat variables (S1 Table). In this assessment of human disturbance, variables such as elevation and vegetation type were included to account for environmental gradients and habitat preferences and are not discussed further, as they are not the focus on this research. In the combined data (all PAs), gray fox, and ringtail occupancies had significant positive associations with road distance; as distance to the nearest road increased, occupancy also increased (Table 1). Rare carnivore occupancy was also positively related to distance to road; however, this relationship varied with PA (Table 1). Rare carnivore occupancy was positively associated with road distance in CHIR (Table 2), while the relationship was non-significant in the other two PAs (Tables 3 and 4). Conversely, coyote occupancy had a negative relationship with road distance (Table 1). In CHIR, mountain lions and ringtail occupancies were positively associated with distance to road (Table 2). Gray fox occupancy was positively related to road distance in ORPI (Table 3). Gray fox occupancy was negatively related to road use overall, but the relationship varied by PA (Table 1; Fig 3). In CHIR, gray fox occupancy was positively associated with road use, while in SAGU, it was negatively associated.

**Table 1:** Species-specific beta estimates (SE) from models of carnivore detections for 7 variables related to human disturbance from 120 sites in three protected areas of southern Arizona, 2014–2015. Only human-disturbance variables that appeared in a top model (ΔAIC < 2; S1 Table) were included.

Parameter	Common carnivores	Rare carnivores	Badger	Bobcat	Coyote
psi	1.490 (0.480)*	-0.208 (0.124)	38.750 (7.980)	-4.127 (0.504)	-2.600 (1.300)
Edge^a^	-	0.240 (0.122)*	1.514 (1.105)	-	2.610 (1.180)*
HD^b^	-	**-**	-	1.452 (0.501)**	0.500 (0.180)**
Park^c^	-	-0.842 (0.150)***	-23.113 (2.730)***	0.833 (0.232)***	2.040 (0.880)*
Road Use	0.810 (0.350)*	**-**	-	-	-
Road Dist^d^	0.470 (0.320)	0.520 (0.161)***	-	-	-0.590 (0.170)***
Trail Use	0.480 (0.732)	**-**	-	-0.663 (0.286)*	-
Trail Dist^e^	-0.360 (0.179)*	1.245 (0.183)***	5.833 (1.439)***	-	-
Park*Edge	-	0.701 (0.489)	-	-	-1.210 (0.610)*
Park*HD	-	-	-	-0.657 (0.238)**	**-**
Park*Road Use	-	**-**	-	-	-
Park*Road Dist	-	**-0.376 (0.214)**	-	-	-
	Gray fox	Hooded skunk	Mountain lion	Ringtail	Spotted skunk
Psi	0.305 (0.100)	3.443 (1.760)	0.217 (.085)	-2.001 (0.439)	-1.070 (0.450)
Edge^a^	0.288 (0.123)*	2.857 (1.186)*	-	**5.787 (3.334)**	-
HD^b^	0.344 (0.158)*	-	-	-	-
Park^c^	2.094 (0.631)***	-4.447 (1.494)**	-1.359 (0.637)*	1.332 (0.864)	-0.540 (0.230)*
Road Use	-3.424 (0.799)***	-	-0.552 (0.442)	-	-
Road Dist^d^	0.534 (0.160)***	2.260 (1.800)	-	0.887 (0.278)**	-
Trail Use	-	-	-	-	-5.657 (0.379)***
Trail Dist^e^	7.439 (0.749)***	-	-	-	-
Park*Edge	-0.715 (0.565)	-1.345 (1.053)	-	2.786 (1.383)*	-
Park*HD	-	-	-	**-**	-
Park*Road Use	1.528 (0.395)***	-	-	-	-
Park*Road Dist	0.395 (0.292)	-	-	-0.318 (0.324)	-

Strength of significance is indicated by

*p < 0.05,

**p < 0.01,

***p < 0.001.

Bolded numbers without * were marginally significant at p < 0.1.

^a^ Distance to nearest edge of protected area.

^b^ Human disturbance index (see methods for description).

^c^ Protected area (CHIR, ORPI, or SAGU).

^d^ Distance to nearest road.

^e^ Distance to nearest trail.

**Table 2:** Species-specific beta estimates (SE) from models of carnivore detections for 6 variables from 40 sites in a conservation easement near the Chiricahuas of southern Arizona, 2014–2015. Only variables that appeared in a top model (ΔAIC < 2) were included.

Parameter	Common carnivores	Rare carnivores	Black bear	Bobcat	Coyote	Gray fox
psi	-2.090 (1.230)	-9.110 (1.970)	-8.851 (2.520)	-2.151 (0.607)	-1.850 (1.040)	-10.300 (3.720)
Edge^a^	-	-0.596 (0.712)	-5.612 (2.390)**	-	1.250 (1.080)	-
HD^b^	-	-	-	0.609 (0.280)*	1.013 (0.651)	-
Infra^c^	-6.440 (2.080)**	-	-7.201 (3.050)*	-1.513 (1.587)	-	-8.380 (1.950)***
Road Use	1.020 (0.510)*	-	-	-0.306 (0.228)	-	0.540 (0.250)*
Road Dist^d^	-	**1.109 (0.649)**	-	-0.752 (0.574)	-1.590 (0.660)*	0.880 (1.421)
	Hooded skunk	Mountain lion	Ringtail	Spotted skunk	Striped skunk	
Psi	-1.531 (0.680)	-0.132 (0.426)	-15.610 (4.410)	-18.370 (3.310)	-8.271 (1.220)	
Edge^a^	1.417 (0.706)*	-1.647 (1.585)	-	-	-2.115 (1.286)	
HD^b^	0.433 (0.285)	-	-	-	-0.072 (0.421)	
Infra^c^	-	-	-7.610 (3.770)*	-7.608 (3.769)*	**-4.011 (2.160)**	
Road Use	-	-	-	-	-	
Road Dist^d^	-	1.448 (0.566)*	1.300 (0.550)*	-	-0.822 (1.008)	

Strength of significance is indicated by

*p < 0.05,

**p < 0.01,

***p < 0.001.

Bolded numbers without * were marginally significant at p < 0.1.

^a^ Distance to nearest edge of protected area.

^b^ Human disturbance index (see methods for description).

^c^ Distance to nearest human infrastructure.

^d^ Distance to nearest road.

**Table 3:** Species-specific beta estimates (SE) from models of carnivore detections for 10 variables from 40 sites in Organ Pipe Cactus National Monument in southern Arizona, 2014–2015. Only variables that appeared in a top model (ΔAIC < 2) were included.

Parameter	Common carnivores	Rare carnivores	Badger	Bobcat	Coyote
psi	2.580 (0.370)	0.305 (0.244)	-3.352 (1.835)	5.807 (4.218)	-2.310 (1.140)
BP^a^	-	-	-1.460 (0.681)*	3.591 (1.633)*	-
Edge^b^	-	-	-	-	-
HD^c^	-	-	**1.125 (0.640)**	-	-
Infra^d^	-	-	-	2.276 (0.982)*	-
Road Use	-	-	-	-	-
Road Dist^e^	-	-	**-**	-	-0.270 (0.180)
Trail Use	-	-	**1.791 (0.980)**	0.762 (0.671)	-
Trail Dist^f^	-	1.076 (0.419)*	**-**	-0.900 (0.620)	-
Visitors^g^	-4.880 (3.980)	-	-	-	-
	Gray fox	Hooded skunk	Kit fox	Mountain lion	Spotted skunk
psi	-3.540 (1.180)	-4.020 (0.850)	-0.266 (0.240)	2.494 (0.359)	-0.440 (1.370)
BP^a^	-	-	-	-	0.547 (0.487)
Edge^b^	-	-	-	-	-
HD^c^	0.890 (0.290)**	0.838 (0.784)	-	-5.909 (0.263)***	-
Infra^d^	-	0.288 (0.202)	-	**-8.814 (5.066)**	-
Road Use	-	-	**0.409 (0.210)**	-	-
Road Dist^e^	0.850 (0.280)**	-1.849 (1.631)	0.994 (0.778)	3.883 (4.682)	-
Trail Use	-	-	**-**	**9.479 (5.283)**	**0.840 (0.500)**
Trail Dist^f^	**-0.410 (0.217)**	-	1.830 (0.430)***	5.034 (0.572)***	0.508 (0.431)
Visitors^g^	-2.330 (2.130)	-	-	-4.067 (1.995)*	-

Strength of significance is indicated by

*p < 0.05,

**p < 0.01,

***p < 0.001.

Bolded numbers without * were marginally significant at p < 0.1.

^a^ Index of Border Patrol activity.

^b^ Distance to nearest edge of protected area.

^c^ Human disturbance index (see methods for description).

^d^ Distance to nearest human infrastructure.

^e^ Distance to nearest road.

^f^ Distance to nearest trail.

^g^ Number of visitors to ORPI for the month in which each survey was conducted.

**Table 4:** Species-specific beta estimates (SE) from models of carnivore detections for 7 variables from 40 sites in Saguaro National Park-West in southern Arizona, 2014–2015. Only variables that appeared in a top model (ΔAIC < 2) were included.

Parameter	Common carnivores	Rare carnivores	Badger	Bobcat
psi	29.880 (2.497)	-0.660 (0.460)	53.994 (3.091)	1.460 (1.127)
Edge^a^	-	-	-	-
HD^b^	-	-	-	-1.439 (0.704)*
Road Use	-	-	-	-
Road Dist^c^	-	-	-	-
Trail Use	-	-	-	-
Trail Dist^d^	-15.919 (17.617)	**-6.694 (3.481)**	3.771 (0.264)***	4.803 (5.493)
	Coyote	Gray fox	Ringtail	Spotted skunk
psi	-1.235 (0.801)	5.750 (2.440)	-2.001 (0.439)	-31.060 (3.120)
Edge^a^	-	-	6.540 (2.760)*	-
HD^b^	0.475 (0.364)	-	-	-
Road Use	-	**-0.740 (0.420)**	-	-
Road Dist^c^	-0.390 (0..280)	-	1.050 (0.480)*	-
Trail Use	-	-	**-22.998 (12.105)**	-
Trail Dist^d^	-	-	**-**	-15.919 (3.510)***

Strength of significance is indicated by

*p < 0.05,

***p < 0.001.

Bolded numbers without * were marginally significant at p < 0.1.

^a^ Distance to nearest edge of protected area.

^b^ Human disturbance index (see methods for description).

^c^ Distance to nearest road.

^d^ Distance to nearest trail.

Overall, common carnivore occupancy was negatively related to trail distance, while rare carnivore, badger, and gray fox occupancies were positively related (Table 1). In ORPI, gray fox occupancy was negatively associated with distance to trail, whereas rare carnivores, kit foxes, and mountain lion occupancies were all positively associated (Table 3). In SAGU, spotted skunk occupancy was negatively related to trail distance, and badger occupancy was positively related (Table 4). Bobcat and spotted skunk occupancies were negatively related to trail use (Table 1).

**Fig 4:**
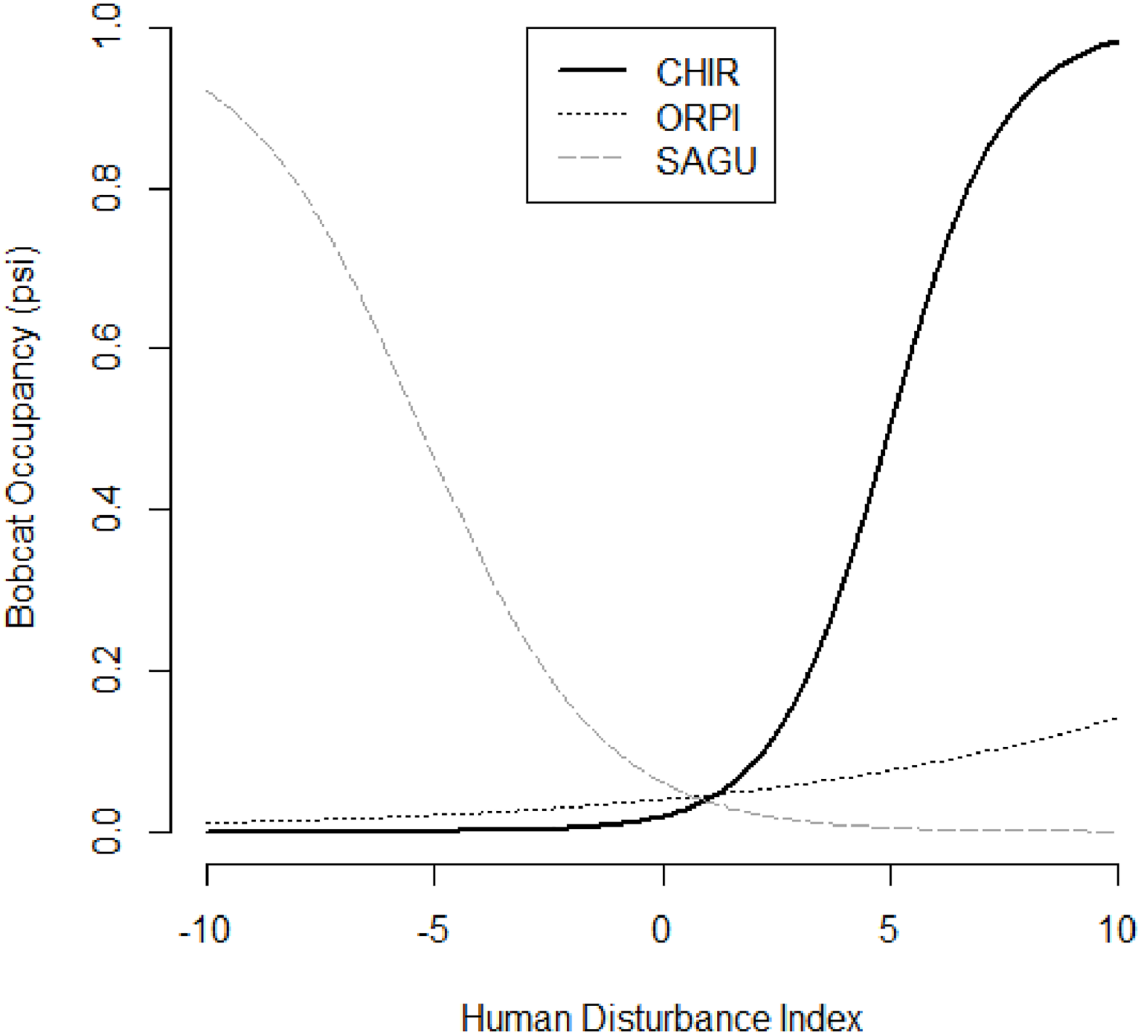
Relationship of bobcat occupancy to an index of human disturbance in 3 protected areas of southern Arizona, 2014–2015.

With data from all the PAs combined, bobcat, coyote, and gray fox occupancies were positively related to the HD index (Table 1). However, the relationship of bobcat occupancy to HD varied among PAs (Fig 4), as it was positively associated in CHIR, while negatively associated in SAGU (Tables 2 and 4). In ORPI, gray fox occupancy was positively related to HD, whereas mountain lion occupancy was negatively related (Table 3). The number of park visitors occurred in the top models of common carnivores, gray foxes, and mountain lions (S1 Table), always negatively associated with occupancy (Table 3). In ORPI, badger occupancy was negatively related to the index of Border Patrol activity (Table 3).

**Fig 5:**
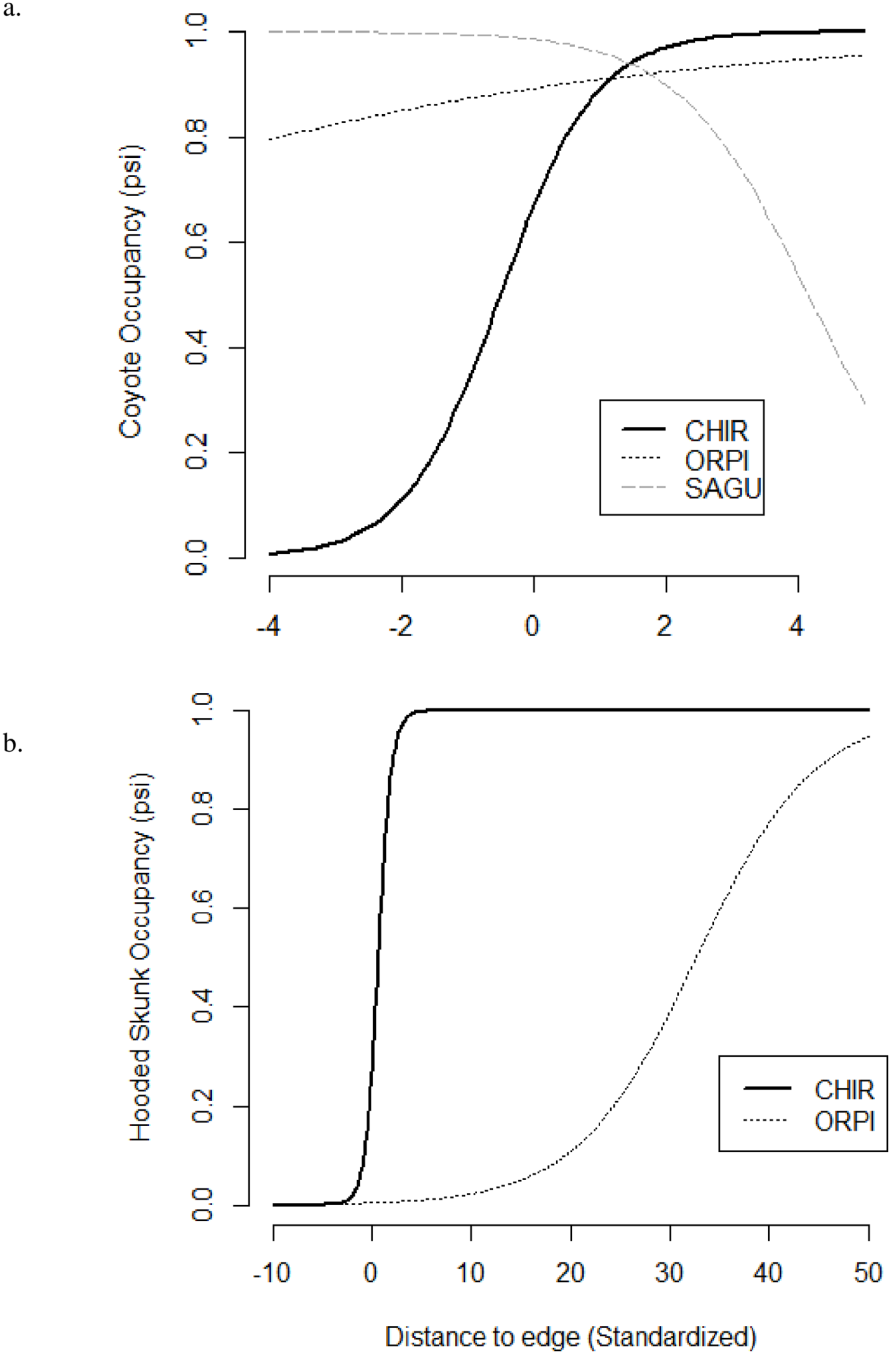
Relationship of a.) coyote and b.) hooded skunk occupancy to distance to edge in 3 protected areas of southern Arizona, 2014–2015.

Occupancies of rare carnivores, coyotes, gray foxes, hooded skunks, and ringtails were positively associated to distance to edge (Table 1; Fig 5); however, the relationship of coyote occupancy to edge distance varied by PA (Fig 5a). In CHIR, black bear (*Ursus americanus*) occupancy was negatively related to distance to edge, and hooded skunk occupancy was positively related (Table 2).

Common carnivore, black bear, gray fox, ringtail and spotted skunk occupancies were all negatively associated with human infrastructure (positively associated with distance) in CHIR (Table 2). In ORPI, however, distance to infrastructure was positively related to occupancy of bobcats (Table 3).

## Discussion

### Carnivore occupancy between PAs

The objective of this study was to understand how humans may influence carnivores in protected areas even when consumptive recreation and extractive uses are not permitted. As hypothesized, human disturbance influenced carnivore occupancies in PAs of southern Arizona, and this effect (whether positive or negative) was species-specific and dependent upon the overall level of disturbance within the PA. Occupancies of nearly all rare carnivore species were higher in PAs with lower levels of disturbance (CHIR and ORPI), while common species had considerably higher occupancy in SAGU (Fig 2). Reed and Merenlender [23] also found that the density of native carnivores was substantially lower in PAs that allowed public access compared to those that did not. Additionally, as predicted, carnivore community diversity was higher in areas with lower levels of disturbance; many rare species were infrequently or never detected in SAGU. It is unlikely that these absent or infrequent detections were a result of low detection probability inherent to the species, as these species were detected readily in other PAs.

### Human disturbance variables

Overall, edges of PAs and the development associated with them appeared to be the most pervasive negative impact of human disturbance on carnivore occupancy as nearly all carnivores had a negative association with edges. Edges of PAs are often a source of negative influence on wildlife species [18]. Crooks [52] also found many carnivore species to be sensitive to habitat fragmentation, including distance to edge. Additionally, mountain lion and leopard use of PAs has been shown to be higher in the interiors of parks [26, 53], and Schuette, Wagner [50] found that distance to human settlement was the most significant component of human disturbance affecting carnivore occupancy in Kenya. In contrast to our study, Reed and Merenlender [23] and Farris, Golden [54] found no indication that edge of PA influenced carnivore distributions.

Roads, a feature common to most PAs and a well-studied aspect of human disturbance, had varying levels of impact on carnivores. Roads positively affected occupancy of coyotes, likely a result of some carnivore’s tendency to use roads for travel [55, 56]. Conversely, most of the rare carnivore species as well as gray foxes had a negative association with roads, a result found in other studies as well [57–59]. These results are in contrast to a study by Davis, Kelley [51] that found that felids were associated with areas of higher road density. Additionally, Lesmeister, Nielsen [49] observed a negative association between bobcats and roads and a positive association between gray foxes and striped skunks (*Mephitis mephitis*) and roads, the opposite of our results. It appears that carnivore responses to roads are dependent on many factors including both species and location.

Gray foxes were the only carnivore to be negatively associated with road use (number of vehicles on the roads), despite the fact that occupancies of most carnivores demonstrated an avoidance of roads (occupancy was positively related to distance from road). Apparently simply the presence of a road, and not necessarily how often it was used, is what most affected carnivores. In southern California, mountain lions showed no discrimination between high and low-speed roads, avoiding both [57]. Additionally, while leopards (*Panthera pardus*) in a PA of Thailand had lower abundance near park roads, the traffic rate did not appear to affect them [26].

It is not surprising that many of the carnivores in this study had a negative association with roads. Mortality due to vehicle collisions represents a serious threat to carnivores [60–62]. Roads can also negatively affect carnivores by modifying and fragmenting the habitat, by presenting obstacles to movement, by changing behavior (e.g., attraction or avoidance), and through noise and visual disturbance [63–65]. Kerley, Goodrich [62] found that PAs appear to no longer operate as a source population for tigers where roads are present.

Trails also had a varying influence on carnivore occupancy. Common carnivores and spotted skunks had a positive association with trails, most likely linked to carnivore use of trails for travelling [66–68]. Most rare carnivore species as well as gray foxes, however, appeared to have a negative association with trails. Many of the same impacts of roads, such as modification and fragmentation of habitat and changes in behavior, can also be seen with trails. Hikers, potentially because their movements are more unpredictable and they are more likely to approach or harass wildlife, elicit a stronger response than vehicles [68]. Hikers on trails often flush wildlife [68, 69], and people on foot may be an even more significant source of disturbance than vehicles [21]. Again, as with roads, few carnivore species were negatively associated with trail use, suggesting that trails negatively impact carnivore occupancy of many species even if they are not well used by people.

Human infrastructure was one of the least important aspects of human disturbance. Infrastructure, such as homes, campgrounds, visitor centers, and other park administration buildings, only had a negative influence on bobcat occupancy. Lesmeister, Nielsen [49] also found that bobcats avoid human infrastructure. Many carnivore species, however, had a positive association with infrastructure in CHIR. This property contained three single family homes with likely small impacts on the environment and carnivores. Even within the national parks, there was little evidence of avoidance of infrastructure by carnivores. While some studies have shown that carnivores avoid areas with human infrastructure [59, 70], these studies were not conducted in PAs. It may be that the infrastructure within PAs is less of a threat than larger scale human development (such as that associated with park edges). Positive associations with human disturbance variables were likely a result of a benefit associated with that variable, such as food resources (garbage), prey vulnerability, or easy travel [71–73].

### Carnivore-specific responses to disturbance

In general, we found coyotes and bobcats to be the carnivores least sensitive to human disturbance within our study areas. Occupancy was high in all PAs, particularly those with higher overall levels of disturbance (SAGU and ORPI). Coyotes were not negatively associated with any variables of human disturbance, except for park edges. Coyotes seem to have the ability to cope with human disturbance compared to other carnivores, which has been demonstrated in many other studies [49, 74–76]. Trail use (overall) and the human disturbance index (in SAGU) were the only negative predictors of bobcat occupancy. While this result is consistent with some studies (e.g., [76]), others have found bobcats to be especially sensitive to human disturbance and development [23, 49, 74, 77].

Gray fox occupancy was one of the most unexpected results in the study. Although their overall occupancy was higher than any other carnivore, specifically in SAGU, they had a number of negative associations with human disturbance variables, including edge, roads and road use, and trails. This differs from many other studies that have found gray foxes to be insensitive to human disturbance [49, 52, 77, 78]. Our results are more aligned with those of Wang, Allen [76] and Ordenana, Crooks [74] which found gray foxes were sensitive to anthropogenic disturbance and development. It is also possible that in these PAs, gray foxes may be avoiding areas such as roads that would put them in contact with coyotes [29, 79].

Smaller carnivores, including ringtails, badgers, and three species of skunks, along with the largest predator in all of the study areas (mountain lion) seem to all be exceptionally vulnerable to human disturbance. These species had the lowest occupancies overall and within the PAs with higher levels of disturbance; many were rarely or never detected in SAGU. Mountain lions, perhaps due to their large home ranges and top trophic position, avoid anthropogenic disturbance and development [52, 57, 74]. Crooks [52] suggested that mustelid/mephitid carnivores are probably more sensitive to disturbance due to their narrower dietary and habitat preferences.

### Human disturbance between PAs

The overall level of human disturbance within each PA affected carnivores’ sensitivity to disturbance variables. Carnivores, with few exceptions, were more sensitive in high disturbance areas (SAGU and ORPI) compared to low disturbance areas (CHIR). Bobcats, for example, were not affected by any human disturbance variables in CHIR and ORPI but had a negative association with the human disturbance index in SAGU (Fig 4). There are several other examples of such associations, where response to disturbance was stronger in high disturbance PAs (see Tables; Fig 5). Frid and Dill [80] assert that human disturbance may be interpreted by animals as a type of predation risk, and that “prey are more vigilant when the perceived risk of predation is greater.” Additionally, King and Workman [81] observed that wildlife reactions to disturbance may be greater when they have been subjected to higher intensities of human activity. This could explain why carnivores responded more strongly to disturbance variables in SAGU and ORPI than CHIR.

### Conclusions

We demonstrate the importance of studying the entire carnivore community, as carnivores exhibited species-specific responses to disturbance. If the goal of protected areas is to maintain an intact carnivore community, the species most sensitive to disturbance should be used as indicators for conservation and management decisions. While area effects have been found to be important for carnivores [18, 52], we were unable to control for area of our PAs as it was confounded with park and visitation. Interestingly and unexpectedly, however, diversity and occupancy were highest for our smallest site (CHIR). Despite its small size, it had the highest diversity of carnivores and the highest occupancy and demonstrated the least impacts of human disturbance. This observation reinforces our conclusion that human activity is likely affecting carnivore communities, and it is possible that disturbance overwhelms the area effects in this environment, although more sites of difference sizes and disturbance levels need to be examined to validate this hypothesis. Kays, Parsons [82] recently suggested that human recreation has relatively minor impacts on most wildlife distributions. However, their study was conducted in forested areas with much denser vegetation compared to the deserts and grasslands of southern Arizona. That the results of our studies differ suggests that there are regional and habitat differences in species’ susceptibility to human disturbance variables [12].

Our study also demonstrates that human disturbance can have significant negative impacts on carnivore occupancy in PAs, particularly those with higher levels of disturbance. Visitors are a major part of the mission of many agencies overseeing PAs and an important source of funding for such agencies. However, the needs of visitors must be properly balanced with the needs of the wildlife within PAs. As the population grows and visitation to PAs continues to increase, levels of human disturbance in southern Arizona PAs will likely also increase in coming years. It is, therefore, essential to create effective management plans which incorporate the impacts of human disturbance within PAs in order to protect these vulnerable and ecologically important species.

## Supporting information

S1 File Description of covariates used for occupancy and detection probabilities. (DOCX)

S1 Table Top ranking (ΔAIC < 2) occupancy models for 11 carnivore species in 3 protected areas of southern Arizona, 2014–2015. (DOCX)

S2 Table Pearson correlation coefficients for all covariates used in the study. (DOCX)
