# Supplementary material for: Impacts of human recreation on carnivores in protected areas
Source: PLoS One. 2018 Apr 5;13(4):e0195436. doi: 10.1371/journal.pone.0195436 (PMC5886570; doi:10.1371/journal.pone.0195436)
Supplement: S1 File — (DOCX) [file pone.0195436.s001.docx]

**Supporting information**

**Occupancy variables**

Distance to Human Infrastructure: Human infrastructure included visitor centers, campgrounds, and houses. The nearest human infrastructure could include a building or house that was outside the protected area (i.e., in SAGU, houses are built near the park edges).

Human Disturbance Index: Information from the surveys and cameras was used to obtain a human disturbance index for each survey site. We counted the number of human influences such as: individual people, domestic animals, and other sign of humans (trash, cow feces, horse tracks, etc.) that were seen by observers or captured on the camera during the 8-day survey session. Trash at each survey site was collected by observers to ensure any trash recorded was new for each survey. To ensure index numbers were conservative, we only counted sign from domestic animals using one method for each survey; for example, if a cow passed by the camera and cow tracks/feces were recorded on the survey, only one of these was counted for the index. We considered the total number of human influences within the survey site during the 8-day survey period as the human disturbance index number for that site; as an example, a site which had two people and a dog pass by the camera (3), a person on a horse seen during a survey (2), and trash (1) found on the first survey day would have an index number of 6.

Road Use: Number of vehicles on each road during the month the survey took place. We then determined the level of use (ranging from 1-4) for each road using percentile rankings (the top 25% percent of roads were given a rating of 4, the next 25% a rating of 3, etc.).

Trail Use: Number of people on each trail during the month the survey took place. We then determined the level of use (ranging from 1-4) for each trail using percentile rankings (the top 25% percent of roads were given a rating of 4, the next 25% a rating of 3, etc.). In SAGU, trail use was not available for all trails from the NPS, so we used information from site surveyors about the number of hikers seen on each trail.

Vegetation Type: Major vegetation type was determined using the USGS Gap Land Cover, hierarchy level ‘Macrogroup’ in ArcGIS.

**Detection probability variables**

Precipitation: Categories for precipitation included: none, light rain, heavy rain, snow.

Human Disturbance Index: The survey-specific human disturbance index was calculated in the same way as the occupancy HD index above, except only the human influences for a single survey were counted.

Temperature: Temperature was excluded as the temperature recorded at the time of each survey was not necessarily related to the temperature when the animal visited the site (we had no way of knowing what the temperature was when a specific track or scat was left at the site).

**Covariate correlations**

We used Pearson correlation tests to determine if variables were correlated. If variables had a statistically significant correlation coefficient where r ≥ 0.50, we did not use those variables within the same models (Table S2). In SAGU models, distance to infrastructure was not used as it significantly correlated with distance to edge (as housing developments surround the park) as well as several other variables. Elevation and distance to edge were also correlated in SAGU. In ORPI, distance to infrastructure was correlated with elevation and trail distance. For all parks combined, distance to infrastructure was significantly correlated with elevation and distance to edge. Number of park visitors and elevation were only used for “within park” comparisons as they were both significantly correlated with “park” (protected area). Major vegetation type and elevation were significantly correlated for all parks.
