# Supplementary material for: Impacts of human recreation on carnivores in protected areas
Source: PLoS One. 2018 Apr 5;13(4):e0195436. doi: 10.1371/journal.pone.0195436 (PMC5886570; doi:10.1371/journal.pone.0195436)
Supplement: S1 Table — (DOCX) [file pone.0195436.s002.docx]

Supplemental Table 1. Top ranking (ΔAIC < 2) occupancy models for 11 carnivore species in 3 protected areas of southern Arizona, 2014-2015.

| Species | Park | Model* | AIC** | Delta AIC | AIC  wt | Model Likelihood | K |
| --- | --- | --- | --- | --- | --- | --- | --- |
| Common carnivores | All | Ψ(RdUse+TrDst), p(.) | 2363.74 | 0.00 | 0.25 | 1.00 | 4 |
|  |  | Ψ(RdDst+RdUse), p(.) | 2363.87 | 0.13 | 0.24 | 0.94 | 4 |
|  |  | Ψ(RdDst+RdUse+TrDst), p(.) | 2364.11 | 0.37 | 0.21 | 0.83 | 5 |
|  |  | Ψ(RdDst+RdUse+TrDst+TrUse), p(.) | 2365.58 | 1.84 | 0.10 | 0.40 | 6 |
|  | CHIR | Ψ(Infra+RdUse), p(.) | 299.64 | 0.00 | 0.49 | 1.00 | 4 |
|  |  | Ψ(.), p(.) | 301.40 | 1.76 | 0.20 | 0.41 | 2 |
|  |  | Ψ(Elev+Infra+RdUse), p(.) | 301.53 | 1.89 | 0.19 | 0.39 | 5 |
|  | ORPI | Ψ(.), p(Prec) | 262.13 | 0.00 | 0.37 | 1.00 | 3 |
|  |  | Ψ(Elev), p(Prec) | 262.76 | 0.63 | 0.27 | 0.73 | 4 |
|  | SAGU | Ψ(.), p(.) | 131.69 | 0.00 | 0.71 | 1.00 | 2 |
| Rare carnivores | All | Ψ(Edge+RdDst+TrDst+Park), p(Prec) | 1528.04 | 0.00 | 0.26 | 1.00 | 6 |
|  |  | Ψ(Edge+RdDst+TrDst), p(Prec) | 1528.17 | 0.13 | 0.24 | 0.94 | 8 |
|  |  | Ψ(Edge+RdDst+TrDst+Park+Park*RdDst), p(Prec) | 1528.34 | 0.30 | 0.22 | 0.86 | 9 |
|  |  | Ψ(Edge+RdDst+TrDst+Park+Park*RdDst+Park*Edge), p(Prec) | 1529.89 | 1.85 | 0.10 | 0.40 | 5 |
|  | CHIR | Ψ(Elev), p(Prec) | 300.95 | 0.00 | 0.36 | 1.00 | 4 |
|  |  | Ψ(Elev+RdDst), p(Prec) | 301.10 | 0.15 | 0.33 | 0.93 | 5 |
|  |  | Ψ(Edge+Elev+RdDst), p(Prec) | 302.77 | 1.82 | 0.14 | 0.40 | 6 |
|  | ORPI | Ψ(TrDst), p(Prec) | 192.29 | 0.00 | 0.42 | 1.00 | 4 |
|  |  | Ψ(Elev+TrDst), p(Prec) | 193.19 | 0.90 | 0.27 | 0.64 | 5 |
|  | SAGU | Ψ(.), p(Prec) | 99.68 | 0.00 | 0.52 | 1.00 | 2 |
| Badger | All | Ψ(Park), p(Prec+Su+W) | 145.73 | 0.00 | 0.31 | 1.00 | 6 |
|  |  | Ψ(Park+Edge), p(Prec+Su+W) | 147.16 | 1.43 | 0.15 | 0.49 | 7 |
|  |  | Ψ(Edge), p(Prec+Su+W) | 147.38 | 1.65 | 0.14 | 0.44 | 6 |
|  |  | Ψ(Park+TrDst), p(Prec+Su+W) | 147.40 | 1.67 | 0.14 | 0.43 | 7 |
|  | ORPI | Ψ(TrUse+BP+HD), p(.) | 126.34 | 0.00 | 0.35 | 1.00 | 5 |
|  |  | Ψ(TrUse+HD), p(.) | 127.70 | 1.36 | 0.18 | 0.51 | 4 |
|  |  | Ψ(TrUse+BP), p(.) | 128.23 | 1.89 | 0.13 | 0.39 | 4 |
|  |  | Ψ(TrUse), p(.) | 128.28 | 1.94 | 0.13 | 0.38 | 3 |
|  | SAGU | Ψ(TrDst), p(Su) | 107.51 | 0.00 | 0.45 | 1.00 | 4 |
| Black bear | CHIR | Ψ(Edge+Infra), p(.) | 51.49 | 0.00 | 0.28 | 1.00 | 4 |
|  |  | Ψ(.), p(.) | 52.55 | 1.06 | 0.17 | 0.59 | 2 |
|  |  | Ψ(Edge), p(.) | 52.56 | 1.07 | 0.17 | 0.59 | 3 |
|  |  | Ψ(Edge+Elev+Infra), p(.) | 52.66 | 1.17 | 0.16 | 0.56 | 5 |
|  |  | Ψ(Infra), p(.) | 53.16 | 1.67 | 0.12 | 0.43 | 3 |
| Bobcat | All | Ψ(TrUse+Park), p(.) | 602.46 | 0.00 | 0.15 | 1.00 | 4 |
|  |  | Ψ(Park), p(.) | 602.52 | 0.06 | 0.15 | 0.97 | 3 |
|  |  | Ψ(HD+TrUse+Park+Park*HD), p(.) | 602.61 | 0.15 | 0.14 | 0.93 | 6 |
|  |  | Ψ(HD+TrUse+Park+Park*HD), p(.) | 602.62 | 0.16 | 0.14 | 0.92 | 7 |
|  |  | Ψ(HD+Park+Park*HD), p(.) | 603.00 | 0.54 | 0.12 | 0.76 | 5 |
|  |  | Ψ(HD+Park), p(.) | 603.81 | 1.35 | 0.08 | 0.51 | 4 |
|  |  | Ψ(HD+TrUse+Park), p(.) | 604.34 | 1.88 | 0.06 | 0.39 | 6 |
|  | CHIR | Ψ(HD), p(.) | 337.91 | 0.00 | 0.20 | 1.00 | 3 |
|  |  | Ψ(HD+RdUse), p(.) | 338.05 | 0.14 | 0.19 | 0.93 | 4 |
|  |  | Ψ(HD+RdDst+RdUse), p(.) | 338.09 | 0.18 | 0.19 | 0.91 | 5 |
|  |  | Ψ(HD+RdDst), p(.) | 338.92 | 1.01 | 0.12 | 0.60 | 4 |
|  |  | Ψ(HD+Infra+RdDst+RdUse), p(.) | 339.17 | 1.26 | 0.11 | 0.53 | 6 |
|  | ORPI | Ψ(Elev+BP), p(.) | 328.21 | 0.00 | 0.24 | 1.00 | 5 |
|  |  | Ψ(Infra+BP), p(.) | 328.51 | 0.30 | 0.21 | 0.86 | 4 |
|  |  | Ψ(Elev+TrDst+BP), p(.) | 328.84 | 0.63 | 0.18 | 0.73 | 6 |
|  |  | Ψ(Elev+TrDst+BP+TrUse), p(.) | 329.68 | 1.47 | 0.12 | 0.48 | 7 |
|  | SAGU | Ψ(Elev+HD), p(.). | 215.42 | 0.00 | 0.29 | 1.00 | 4 |
|  |  | Ψ(Elev), p(.). | 216.37 | 0.95 | 0.18 | 0.62 | 3 |
|  |  | Ψ(.), p(.) | 216.43 | 1.01 | 0.17 | 0.60 | 2 |
|  |  | Ψ(HD), p(.). | 216.70 | 1.28 | 0.15 | 0.53 | 3 |
|  |  | Ψ(Elev+HD+TrDst), p(.). | 216.98 | 1.56 | 0.13 | 0.46 | 5 |
| Coyote | All | Ψ(Edge+HD+RdDst+Park+Park*Edge), p(.) | 2243.17 | 0.00 | 0.77 | 1.00 | 7 |
|  | CHIR | Ψ(HD+RdDst), p(.) | 349.80 | 0.00 | 0.37 | 1.00 | 4 |
|  |  | Ψ(RdDst), p(.) | 350.81 | 1.01 | 0.27 | 0.60 | 3 |
|  |  | Ψ(Edge+HD+RdDst), p(.) | 350.87 | 1.07 | 0.22 | 0.59 | 5 |
|  | ORPI | Ψ(Elev), p(.) | 188.75 | 0.00 | 0.38 | 1.00 | 4 |
|  |  | Ψ(.), p(.) | 189.84 | 1.09 | 0.22 | 0.58 | 3 |
|  |  | Ψ(Elev+RdDst), p(.) | 190.38 | 1.63 | 0.17 | 0.44 | 5 |
|  | SAGU | Ψ(Elev), p(.) | 449.81 | 0.00 | 0.34 | 1.00 | 3 |
|  |  | Ψ(Elev+HD), p(.) | 450.66 | 0.85 | 0.22 | 0.65 | 4 |
|  |  | Ψ(Elev+RdDst), p(.) | 450.74 | 0.93 | 0.21 | 0.63 | 4 |
|  |  | Ψ(Elev+HD+RdDst), p(.) | 451.66 | 1.85 | 0.13 | 0.40 | 5 |
| Gray fox | All | Ψ(Edge+HD+RdDst+RdUse+TrDst+Park+Park*RdUse), p(.) | 2276.00 | 0.00 | 1.00 | 1.00 | 10 |
|  | CHIR | Ψ(Elev+Infra+RdUse), p(.) | 296.38 | 0.00 | 0.60 | 1.00 | 5 |
|  |  | Ψ(Elev+Infra+RdDst+RdUse), p(.) | 298.19 | 1.81 | 0.24 | 0.40 | 6 |
|  | ORPI | Ψ(Elev+HD+RdDst), p(.) | 291.87 | 0.00 | 0.34 | 1.00 | 5 |
|  |  | Ψ(Elev+HD+RdDst+TrDst), p(.) | 292.35 | 0.48 | 0.27 | 0.79 | 6 |
|  |  | Ψ(Elev+HD+RdDst+Visitors), p(.) | 293.43 | 1.56 | 0.16 | 0.46 | 6 |
|  |  | Ψ(Elev+HD+RdDst+TrDst+Visitors), p(.) | 293.82 | 1.95 | 0.13 | 0.38 | 7 |
|  | SAGU | Ψ(Elev+RdUse), p(.) | 358.87 | 0.00 | 0.28 | 1.00 | 4 |
|  |  | Ψ(Elev), p(.) | 359.00 | 0.13 | 0.26 | 0.94 | 3 |
|  |  | Ψ(.), p(.) | 359.48 | 0.61 | 0.21 | 0.74 | 2 |
|  |  | Ψ(Elev+RdUse+TrUse), p(.) | 360.05 | 1.18 | 0.15 | 0.55 | 5 |
| Hooded skunk | All | Ψ(Edge+Park), p(.) | 301.26 | 0.00 | 0.26 | 1.00 | 4 |
|  |  | Ψ(Edge+Park+Park*Edge), p(.) | 301.63 | 0.37 | 0.22 | 0.83 | 5 |
|  |  | Ψ(Park), p(.) | 301.64 | 0.38 | 0.22 | 0.83 | 3 |
|  |  | Ψ(Edge+RdDst+Park), p(.) | 302.79 | 1.53 | 0.12 | 0.47 | 5 |
|  |  | Ψ(Edge+RdDst+Park+Park*Edge), p(.) | 303.19 | 1.93 | 0.10 | 0.38 | 6 |
|  | CHIR | Ψ(Edge+HD), p(.) | 297.61 | 0.00 | 0.28 | 1.00 | 4 |
|  |  | Ψ(Edge), p(.) | 297.95 | 0.34 | 0.24 | 0.84 | 3 |
|  |  | Ψ(Edge+Elev+HD), p(.) | 299.02 | 1.41 | 0.14 | 0.49 | 5 |
|  |  | Ψ(HD), p(.) | 299.31 | 1.70 | 0.12 | 0.43 | 3 |
|  |  | Ψ(.), p(.) | 299.47 | 1.86 | 0.11 | 0.39 | 2 |
|  | ORPI | Ψ(.), p(.) | 33.97 | 0.00 | 0.23 | 1.00 | 2 |
|  |  | Ψ(Elev), p(.) | 34.87 | 0.90 | 0.14 | 0.64 | 3 |
|  |  | Ψ(Elev+RdDst), p(.) | 34.89 | 0.92 | 0.14 | 0.63 | 4 |
|  |  | Ψ(Infra), p(.) | 35.09 | 1.12 | 0.13 | 0.57 | 3 |
|  |  | Ψ(HD), p(.) | 35.61 | 1.64 | 0.10 | 0.44 | 3 |
| Kit fox | ORPI | Ψ(TrDst), p(Prec) | 117.02 | 0.00 | 0.40 | 1.00 | 4 |
|  |  | Ψ(RdUse+TrDst), p(Prec) | 118.12 | 1.10 | 0.23 | 0.58 | 5 |
|  |  | Ψ(RdDst+RdUse+TrDst), p(Prec) | 118.35 | 1.33 | 0.21 | 0.51 | 6 |
| Mountain lion | All | Ψ(Park), p(.) | 102.75 | 0.00 | 0.39 | 1.00 | 3 |
|  |  | Ψ(.), p(.) | 103.39 | 0.64 | 0.28 | 0.73 | 2 |
|  |  | Ψ(RdUse), p(.) | 104.30 | 1.55 | 0.18 | 0.46 | 3 |
|  |  | Ψ(RdUse+Park), p(.) | 104.57 | 1.82 | 0.16 | 0.40 | 4 |
|  | CHIR | Ψ(RdDst), p(.) | 62.75 | 0.00 | 0.35 | 1.00 | 3 |
|  |  | Ψ(.), p(.) | 63.00 | 0.25 | 0.31 | 0.88 | 2 |
|  |  | Ψ(Edge+RdDst), p(.) | 64.22 | 1.47 | 0.17 | 0.48 | 4 |
|  | ORPI | Ψ(Elev+HD+Visitors), p(.) | 51.08 | 0.00 | 0.17 | 1.00 | 5 |
|  |  | Ψ(Elev+HD), p(.) | 52.13 | 1.05 | 0.10 | 0.59 | 4 |
|  |  | Ψ(Visitors), p(.) | 52.27 | 1.19 | 0.09 | 0.55 | 3 |
|  |  | Ψ(TrDst), p(.) | 52.27 | 1.19 | 0.09 | 0.55 | 3 |
|  |  | Ψ(TrUse), p(.) | 52.55 | 1.47 | 0.08 | 0.48 | 3 |
|  |  | Ψ(.), p(.) | 52.58 | 1.50 | 0.08 | 0.47 | 2 |
|  |  | Ψ(Infra), p(.) | 52.66 | 1.58 | 0.08 | 0.45 | 3 |
|  |  | Ψ(Elev), p(.) | 52.66 | 1.58 | 0.08 | 0.45 | 3 |
|  |  | Ψ(RdDst), p(.) | 52.78 | 1.70 | 0.07 | 0.43 | 3 |
| Ringtail | All | Ψ(RdDst), p(.) | 206.09 | 0.00 | 0.29 | 1.00 | 3 |
|  |  | Ψ(Edge+RdDst+Park+Park*Edge), p(.) | 206.45 | 0.36 | 0.24 | 0.84 | 6 |
|  |  | Ψ(RdDst+Park), p(.) | 207.48 | 1.39 | 0.14 | 0.50 | 4 |
|  |  | Ψ(Edge+RdDst+Park+Park*Edge+Park*RdDst), p(.) | 207.48 | 1.39 | 0.14 | 0.50 | 7 |
|  |  | Ψ(RdDst+Edge), p(.) | 208.08 | 1.99 | 0.11 | 0.37 | 4 |
|  | CHIR | Ψ(Elev+Infra), p(.) | 88.80 | 0.00 | 0.50 | 1.00 | 4 |
|  |  | Ψ(Elev+Infra+RdDst), p(.) | 90.75 | 1.95 | 0.19 | 0.38 | 5 |
|  | SAGU | Ψ(Edge+RdDst), p(.) | 102.80 | 0.00 | 0.40 | 1.00 | 4 |
|  |  | Ψ(Edge+RdDst+TrUse), p(.) | 103.60 | 0.80 | 0.27 | 0.67 | 5 |
| Spotted skunk | All | Ψ(Park), p(.) | 183.85 | 0.00 | 0.35 | 1.00 | 3 |
|  |  | Ψ(.), p(.) | 184.20 | 0.35 | 0.29 | 0.84 | 2 |
|  |  | Ψ(Park+TrUse), p(.) | 185.58 | 1.73 | 0.15 | 0.42 | 4 |
|  | CHIR | Ψ(Elev), p(.) | 70.44 | 0.00 | 0.46 | 1.00 | 3 |
|  |  | Ψ(Elev+Infra), p(.) | 71.94 | 1.50 | 0.22 | 0.47 | 4 |
|  | ORPI | Ψ(Elev+TrUse), p(.) | 123.17 | 0.00 | 0.30 | 1.00 | 4 |
|  |  | Ψ(Elev), p(.) | 123.64 | 0.47 | 0.24 | 0.79 | 3 |
|  |  | Ψ(Elev+TrDst+TrUse), p(.) | 123.91 | 0.74 | 0.21 | 0.69 | 5 |
|  |  | Ψ(Elev+TrDst+TrUse+BP), p(.) | 124.63 | 1.46 | 0.14 | 0.48 | 6 |
|  | SAGU | Ψ(Elev+TrDst), p(.) | 52.19 | 0.00 | 0.30 | 1.00 | 4 |
|  |  | Ψ(Elev), p(.) | 52.35 | 0.16 | 0.28 | 0.92 | 3 |
|  |  | Ψ(TrDst), p(.) | 52.80 | 0.61 | 0.22 | 0.74 | 3 |
|  |  | Ψ(.), p(.) | 53.13 | 0.94 | 0.19 | 0.63 | 2 |
| Striped skunk | CHIR | Ψ(Elev), p(.) | 207.70 | 0.00 | 0.27 | 1.00 | 3 |
|  |  | Ψ(Elev+Edge+Infra), p(.) | 207.90 | 0.20 | 0.25 | 0.90 | 5 |
|  |  | Ψ(Elev+Infra), p(.) | 208.12 | 0.42 | 0.22 | 0.81 | 4 |
|  |  | Ψ(Elev+Edge), p(.) | 209.17 | 1.47 | 0.13 | 0.48 | 4 |

* Variable descriptions: BP = Border Patrol index of activity, Edge = distance to nearest edge of protected area, Elev = elevation at the sample site, HD = index of human disturbance (see supplemental methods for details), Infra = distance to nearest human infrastructure, Park = protected area, RdDst = distance to nearest road, RdUse = categorical measure of the amount of traffic on the nearest road, TrDst = distance to nearest trail, TrUse = categorical measure of the amount of hikers using the nearest trail, Visitors = number of visitors during the month in which the survey took place, Prec = level of precipitation.

** QAIC was used when ĉ > 1: common carnivores (CHIR, ORPI, SAGU), rare carnivores (CHIR, ORPI, SAGU), coyote (CHIR, ORPI, SAGU), gray fox (CHIR, ORPI, SAGU), kit fox (ORPI), spotted skunk (All, CHIR, SAGU), black bear (CHIR), mountain lion (All, CHIR), bobcat (All, ORPI, SAGU)
