# Supplementary material for: Impacts of human recreation on carnivores in protected areas
Source: PLoS One. 2018 Apr 5;13(4):e0195436. doi: 10.1371/journal.pone.0195436 (PMC5886570; doi:10.1371/journal.pone.0195436)
Supplement: S2 Table — (DOCX) [file pone.0195436.s003.docx]

Supplemental Table 2. Pearson correlation coefficients for all covariates used in the study.

A. All park data combined, B. CHIR, C. ORPI, and D. SAGU.

| A. | Edge | Elevation | HD | Infra | RdDst | RdUse | TrDst | TrUse | Season | Park | Visitors | VegType |
| --- | --- | --- | --- | --- | --- | --- | --- | --- | --- | --- | --- | --- |
| Edge |  | -0.33 | 0.03 | 0.61* | 0.22 | -0.08 | 0.15 | -0.15 | -0.01 | -0.02 | -0.16 | -0.22 |
| Elevation |  |  | 0.07 | -0.50* | -0.37 | -0.01 | 0.21 | -0.15 | 0.00 | -0.70* | 0.01 | -0.91* |
| HD |  |  |  | 0.00 | -0.06 | 0.04 | 0.06 | -0.06 | -0.12 | -0.14 | -0.15 | -0.08 |
| Infra |  |  |  |  | 0.14 | -0.09 | 0.46 | -0.21 | -0.02 | -0.02 | -0.14 | -0.14 |
| RdDst |  |  |  |  |  | -0.10 | -0.16 | 0.09 | -0.02 | 0.27 | 0.21 | -0.12 |
| RdUse |  |  |  |  |  |  | -0.05 | -0.01 | 0.00 | 0.01 | 0.01 | 0.01 |
| TrDst |  |  |  |  |  |  |  | -0.37 | -0.01 | -0.29 | -0.03 | 0.16 |
| TrUse |  |  |  |  |  |  |  |  | 0.02 | 0.18 | -0.02 | -0.17 |
| Season |  |  |  |  |  |  |  |  |  | -0.01 | 0.00 | 0.00 |
| Park |  |  |  |  |  |  |  |  |  |  | 0.98* | -0.35 |
| Visitors |  |  |  |  |  |  |  |  |  |  |  | -0.29 |
|  |  |  |  |  |  |  |  |  |  |  |  |  |
| B. | Edge | Elevation | HD | Infra | RdDst | RdUse | Season | VegType |  |  |  |  |
| Edge |  | 0.25 | -0.01 | -0.33 | -0.01 | -0.19 | 0.01 | -0.04 |  |  |  |  |
| Elevation |  |  | -0.11 | 0.03 | 0.14 | 0.03 | -0.01 | 0.28 |  |  |  |  |
| HD |  |  |  | -0.09 | -0.19 | 0.03 | -0.04 | -0.02 |  |  |  |  |
| Infra |  |  |  |  | -0.18 | 0.24 | 0.02 | -0.01 |  |  |  |  |
| RdDst |  |  |  |  |  | -0.25 | -0.06 | 0.17 |  |  |  |  |
| RdUse |  |  |  |  |  |  | 0.05 | 0.10 |  |  |  |  |
| Season |  |  |  |  |  |  |  | -0.02 |  |  |  |  |

|  |  |  |  |  |  |  |  | |  |  |  |  |  |
| --- | --- | --- | --- | --- | --- | --- | --- | --- | --- | --- | --- | --- | --- |
| C. | BP | Edge | Elevation | HD | Infra | RdDst | RdUse | | TrDst | TrUse | Season | Visitors | VegType |
| BP |  | -0.30 | 0.36 | 0.30 | -0.32 | -0.08 | 0.04 | | -0.29 | 0.38 | -0.01 | -0.01 | 0.01 |
| Edge |  |  | -0.21 | -0.10 | 0.28 | -0.08 | | -0.13 | 0.00 | -0.27 | -0.03 | -0.02 | 0.00 |
| Elevation |  |  |  | 0.12 | -0.82* | 0.07 | 0.19 | | -0.22 | 0.36 | 0.04 | 0.02 | 0.72* |
| HD |  |  |  |  | -0.07 | 0.02 | 0.04 | | -0.08 | 0.08 | -0.17 | -0.25 | 0.00 |
| Infra |  |  |  |  |  | -0.14 | -0.15 | | 0.90* | -0.46 | -0.07 | -0.04 | -0.02 |
| RdDst |  |  |  |  |  |  | -0.11 | | -0.10 | 0.12 | -0.01 | -0.02 | -0.01 |
| RdUse |  |  |  |  |  |  |  | | -0.06 | 0.12 | 0.01 | -0.01 | 0.06 |
| TrDst |  |  |  |  |  |  |  | |  | -0.33 | -0.03 | -0.02 | 0.00 |
| TrUse |  |  |  |  |  |  |  | |  |  | 0.10 | -0.01 | -0.04 |
| Season |  |  |  |  |  |  |  | |  |  |  | -0.14 | 0.00 |
| Visitors |  |  |  |  |  |  |  | |  |  |  |  | -0.14 |
|  |  |  |  |  |  |  |  | |  |  |  |  |  |
| D. | Edge | Elevation | HD | Infra | RdDst | RdUse | TrDst | | TrUse | Season | Visitors | VegType |  |
| Edge |  | 0.66* | -0.05 | 0.50* | 0.14 | -0.15 | -0.34 | | -0.07 | 0.00 | 0.00 | -0.25 |  |
| Elevation |  |  | -0.10 | 0.55* | 0.24 | -0.16 | -0.25 | | 0.13 | -0.01 | 0.00 | -0.63* |  |
| HD |  |  |  | -0.11 | -0.09 | 0.15 | -0.01 | | -0.02 | -0.18 | 0.01 | 0.09 |  |
| Infra |  |  |  |  | 0.18 | -0.07 | -0.32 | | -0.18 | 0.00 | -0.01 | -0.55* |  |
| RdDst |  |  |  |  |  | 0.13 | -0.12 | | 0.05 | -0.01 | 0.03 | -0.07 |  |
| RdUse |  |  |  |  |  |  | -0.11 | | 0.2 | -0.01 | -0.08 | -0.15 |  |
| TrDst |  |  |  |  |  |  |  | | -0.22 | -0.02 | 0.04 | 0.07 |  |
| TrUse |  |  |  |  |  |  |  | |  | 0.02 | -0.03 | 0.16 |  |
| Season |  |  |  |  |  |  |  | |  |  | 0.30 | 0.03 |  |
| Visitors |  |  |  |  |  |  |  | |  |  |  | 0.03 |  |

Variable descriptions: BP = Border Patrol index of activity, Edge = distance to nearest edge of protected area, Elevation = elevation at the sample site, HD = index of human disturbance (see supplemental methods for details), Infra = distance to nearest human infrastructure, Park = protected area, RdDst = distance to nearest road, RdUse = categorical measure of the amount of traffic on the nearest road, TrDst = distance to nearest trail, TrUse = categorical measure of the amount of hikers using the nearest trail, Visitors = number of visitors during the month in which the survey took place, VegType = major vegetation type at the sample site using USGS Gap Land Cover, hierarchy level ‘Macrogroup’

*Indicates statistical significance (p < 0.05).
